# Untargeted longitudinal analysis of a wellness cohort identifies markers of metastatic cancer years prior to diagnosis

**DOI:** 10.1038/s41598-020-73451-z

**Published:** 2020-10-01

**Authors:** Andrew T. Magis, Noa Rappaport, Matthew P. Conomos, Gilbert S. Omenn, Jennifer C. Lovejoy, Leroy Hood, Nathan D. Price

**Affiliations:** 1 Institute for Systems Biology, 401 Terry Ave N, Seattle, WA 98109 USA; 2 Department of Biostatistics, University of Washington, Seattle, WA USA; 3 Departments of Computational Medicine & Bioinformatics, Internal Medicine, Human Genetics, and School of Public Health, University of Michigan, Ann Arbor, MI USA; 4 Providence St. Joseph Health, Seattle, WA USA

**Keywords:** Diagnostic markers, Predictive markers, Cancer screening, Time series

## Abstract

We analyzed 1196 proteins in longitudinal plasma samples from participants in a commercial wellness program, including samples collected pre-diagnosis from ten cancer patients and 69 controls. For three individuals ultimately diagnosed with metastatic breast, lung, or pancreatic cancer, CEACAM5 was a persistent longitudinal outlier as early as 26.5 months pre-diagnosis. CALCA, a biomarker for medullary thyroid cancer, was hypersecreted in metastatic pancreatic cancer at least 16.5 months pre-diagnosis. ERBB2 levels spiked in metastatic breast cancer between 10.0 and 4.0 months pre-diagnosis. Our results support the value of deep phenotyping seemingly healthy individuals in prospectively inferring disease transitions.

## Introduction

A central premise of predictive, preventive, personalized, and participatory (P4) medicine^1^ is the identification of early markers of disease transitions. One important example is identification of cancers prior to metastasis, enabling localized treatment options to remove the primary cancer, improving survival and reducing probability of recurrence. Multiple such biomarker approaches are being studied, including circulating tumor cells^2^, cell-free DNA^3^, and circulating proteins^4^.

Studies seeking to identify cancer biomarkers typically compare samples derived from diagnosed cancer patients with samples derived from non-cancer controls. One recent study leveraging this approach identified a predictive set of protein biomarkers for eight common cancer types^5^. Of greater potential to identify early biomarkers for cancer is the analysis of pre-diagnosis samples from seemingly healthy individuals, later diagnosed with cancer. Opportunities to do this are rare, but can yield insights into early signals and mechanisms of disease transitions. For this study, we analyzed pre-diagnosis plasma samples collected at regular intervals from ‘healthy’ individuals participating in a commercial scientific wellness program (Arivale, Inc)^6^ (Supplementary Table S1).

## Results

Individuals self-reported any experience of a hospitalization or serious disease diagnosis while participating in the scientific wellness program. Over three years, ten individuals with at least three biobanked plasma samples reported a cancer diagnosis (Table 1) (cases). Each of these 37 samples preceded an individual’s diagnosis by 2.5–29.5 months, with *μ* = 168 days between samples. An additional 210 samples were analyzed concurrently, collected from 69 individuals with at least three longitudinal samples (*μ* = 163 days between samples) who did not report a cancer diagnosis over this period (controls). We used the Olink platform^7^ to measure the pre-diagnosis abundance of 1196 proteins (Supplementary Table S2).

**Table 1:** Cancer types in this study with three or more plasma samples preceding the diagnosis.

Cancer type	Sex	Age (baseline)	# Draws before diagnosis	Earliest draw before diagnosis (days)	Latest draw before diagnosis (days)	Mean time between draws (days)
Pancreatic cancer, stage 4	F	57	4	− 502	− 103	133.0
Breast cancer, stage 4	F	56	3	− 423	− 122	150.5
Lung cancer, stage 4	M	69	4	− 803	− 198	201.7
Chronic lymphocytic leukemia	M	68	3	− 346	− 88	129.0
Chronic myeloid leukemia	M	75	4	− 741	− 179	187.3
Bladder cancer, stage 3	F	64	4	− 742	− 138	201.3
Prostate cancer	M	64	3	− 316	− 75	120.5
Prostate cancer	M	48	4	− 775	− 206	189.7
Melanoma	M	67	3	− 424	− 109	157.5
Melanoma	F	49	5	− 901	− 207	173.5

**Figure 1:**
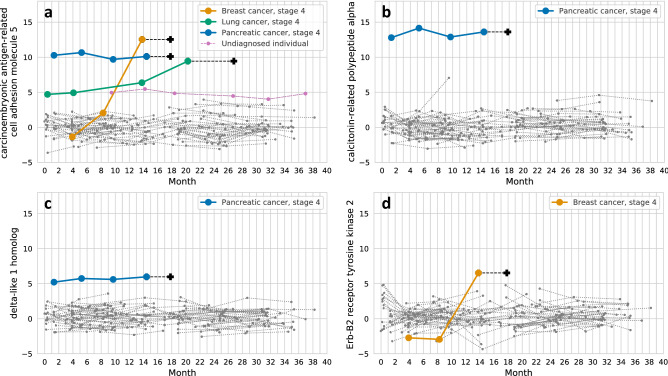
Longitudinal trajectories of selected outlier proteins across multiple cancer types in individuals. Plus (+) signs indicate the diagnosis date (if applicable) for that disease trajectory. Y-axis values are median absolute deviation (MAD). Unlabeled trajectories (grey lines) represent trajectories for all other samples in the study for that protein. (**a**) CEACAM5 was a persistent outlier in pre-diagnosis samples for two metastatic cancer individuals (lung and pancreatic) and exhibited rapid change to extreme outlier levels for metastatic breast cancer. One undiagnosed individual with skin lesions also exhibited elevated levels and fluctuating outlier status for CEACAM5. (**b**) CALCA was a persistent outlier in metastatic pancreatic cancer. (**c**) DLK1 was a persistent outlier in metastatic pancreatic cancer. (**d**) ERBB2 rapidly increased from low levels to outlier levels over a period of six months preceding the diagnosis of metastatic breast cancer.

For all three individuals who were later diagnosed with stage 4 (metastatic) cancers, we observed carcinoembryonic antigen-related cell adhesion molecule 5 (CEACAM5) as an outlier in one or more pre-diagnosis samples (Fig. 1a). CEACAM5 is known to be overexpressed in breast, lung, and pancreatic primary and metastatic tumors^8,9^. For one individual in our study diagnosed with stage 4 pancreatic cancer, CEACAM5 was a persistent outlier (9.7–10.7 MAD) in plasma for four consecutive samples, collected 16.5, 12.5, 8.0, and 3.5 months pre-diagnosis. A second individual diagnosed with metastatic lung cancer was a monotonically-increasing persistent outlier (4.9–9.4 MAD) for CEACAM5 for four plasma samples, collected 26.5, 22.5, 13.0, and 6.5 months pre-diagnosis. A third individual, diagnosed with metastatic breast cancer, exhibited the highest levels of CEACAM5, rapidly increasing from low levels (− 1.4 MAD) 14.0 months pre-diagnosis to extreme outlier levels (12.5 MAD) 4.0 months pre-diagnosis. Resampling demonstrated this protein outlier configuration was significantly rare, and unlikely to have occurred by chance (*p* < 1e−5) (Supplementary Fig. S1). In contrast to the findings in metastatic cancer, CEACAM5 consistently remained below the outlier threshold for all five non-metastatic solid cancer diagnoses and two blood cancers in this study. Importantly, no other measured protein was an outlier specifically across metastatic cancers.

For 68 of the 69 control individuals, CEACAM5 plasma levels consistently remained below the outlier threshold. At the time this study was performed, one undiagnosed individual’s CEACAM5 levels were observed to fluctuate around outlier status (4.0–5.5 MAD) for six consecutive blood draws over a period of 27.5 months (Fig. 1a). Eight months after the final blood collection, this individual reported undergoing treatment for precancerous skin lesions. Unfortunately, no further follow up information is available for this individual, though we note that cutaneous metastases from stage 4 cancers have been reported to mimic benign skin lesions and can be the initial presentation of an underlying malignancy^10^.

We examined other protein outliers besides CEACAM5. Two other persistent outliers for the case of metastatic pancreatic cancer were calcitonin-related polypeptide alpha (CALCA) and delta-like 1 homolog (DLK1) (Fig. 1b,c, Supplementary Fig. S2). Each followed a similar pattern as CEACAM5, remaining outliers throughout the 16.5 months preceding diagnosis. Neither protein was a persistent outlier in any other sample.

In the metastatic breast cancer case, 43 proteins exhibited significantly rapid change in addition to CEACAM5 (Supplementary Fig. S3a–f). Erb-B2 receptor tyrosine kinase 2 (ERBB2, a.k.a. HER2) increased from very low levels (− 2.9 MAD) to very high levels (6.5 MAD) between 10.0 and 4.0 months pre-diagnosis (Fig. 1d). Other proteins with similar dynamics included nectin cell adhesion molecule 4 (NECTIN4) and secreted phosphoprotein 1 (SPP1).

Unsurprisingly, blood cancers exhibited a large number of plasma protein outliers. One individual diagnosed with chronic lymphocytic leukemia was a persistent outlier for 13 proteins across three plasma samples collected 11.5, 8.0, and 3.0 months prior to the diagnosis (Supplementary Fig. S4a,b). We note that this individual also exhibited out-of-range high absolute lymphocyte counts over this period (6.2, 7.9, and 7.1 × 10e3/μL), which ultimately led to the cancer diagnosis following a physician referral by the Arivale coach. Persistent outliers included nucleophosmin (NPM1), T-cell leukemia/lymphoma 1A (TCL1A), lymphocyte-activation gene 3 (LAG3), T-cell surface glycoprotein CD5 (CD5), and T-cell surface glycoprotein CD6 (CD6).

To validate our CEACAM5 observations, we used batch-corrected RNA-seq data derived from The Cancer Genome Atlas (TCGA) Pan-Cancer Initiative^11^ from normal tissue, primary tumor tissue, and metastatic or recurrent tumor tissue. *CEACAM5* expression was significantly higher in primary lung adenocarcinoma (*p* = 1.5e−14), primary breast invasive carcinoma (*p* = 2.1e−11), and primary pancreatic adenocarcinoma (*p* = 3.0e−6) when compared to normal lung, breast, and pancreatic tissues, respectively, adjusting for age and sex. Due to low sample numbers, we were not able to statistically evaluate differences in *CEACAM5* expression for metastatic/recurrent tumor samples, though *CEACAM5* expression in these tissues was qualitatively similar to primary tumor tissues. (Supplementary Fig. S5).

## Discussion

To our knowledge, this is the first study to measure the dynamics of CEACAM5, as well as 1195 other plasma proteins, from samples collected as many as 29.5 months pre-diagnosis of metastatic cancers. Although ten separate trajectories to cancer diagnoses were examined (Table 1), CEACAM5 was observed to be a persistent outlier only in the three cancers each later diagnosed as metastatic, as well as one undiagnosed individual with skin lesions. Importantly, this persistent outlier status was observed across three distinct late stage cancer types: lung, breast, and pancreatic, suggesting pre-diagnosis overexpression of CEACAM5 is not tissue-specific, but rather implicating it with metastasis. Due to the prospective nature of this study, and the fact that the cohort was generally healthy, we were only able to identify a small number of metastatic cancer cases, limiting the generalizability of our findings. More follow-up studies with larger N are clearly needed to validate the clinical utility of these observations, as CEACAM5 represents a promising candidate for early warning of metastasis.

Interpretation of pre-diagnosis trajectories can be contextualized with existing literature evidence to guide prospective decisions to increase surveillance. In the case of metastatic pancreatic cancer, we observed very high levels of a circulating marker of metastasis (CEACAM5), calcitonin hyper-secretion (CALCA), and persistently elevated delta-like 1 homolog (DLK1) at least 16.5 months prior to the diagnosis. Calcitonin is a hormone normally produced by the thyroid that acts to reduce circulating calcium. It is a tumor screening biomarker for medullary thyroid cancer^12^, and is hyper-secreted in other diseases^13^. Calcitonin-secreting neuroendocrine tumors are described in the medical literature^14,15^ and recent guidelines recommend pancreas screening if elevated calcitonin levels are observed in the absence of thyroid cancer^16,17^. DLK1 is widely expressed during embryonic development, whereas postnatal expression is primarily observed in the beta cells of the islets of Langerhans in the adult pancreas^18^. Pancreatic cancer workup and surveillance (or treatment) for this individual could have been justified at least a year prior to the diagnosis had this retrospective protein analysis been performed prospectively^8,9,14–18^. Since all three proteins were already elevated when this individual enrolled in the scientific wellness program, we cannot know how far into the past such a prospective analysis could have been applied. This is particularly important for pancreatic cancers, typically initially discovered at a late, incurable stage.

In the metastatic breast cancer case, CEACAM5 spiked from normal levels to extreme outlier levels between 14.0 and 4.0 months prior to the diagnosis. As CEACAM5 is a clinically-relevant marker of breast cancer metastasis^19^, this dynamic shift suggests metastasis may have been initiated during the 10-month period prior to the diagnosis. Interestingly, we observed 43 other proteins spike over the same time period. Notably, Erb-B2 receptor tyrosine kinase 2 (ERBB2, a.k.a. HER2) increased from very low levels (− 2.9 MAD) to very high levels (6.5 MAD) between 10.0 and 4.0 months pre-diagnosis. Overexpression of ERBB2 has been reported in multiple cancers, including breast, ovarian, and gastric tumors. Similar dynamic change was observed in nectin cell adhesion molecule 4 (NECTIN4) and secreted phosphoprotein 1 (SPP1), which have been identified previously as markers for breast cancer progression or metastasis^20,21^. We propose that concordant dynamic shifts from normal baseline levels across multiple proteins represent understudied signatures of disease transitions that could justify increased surveillance in an N-of-1 paradigm.

To validate our CEACAM5 observations, we obtained batch corrected RNA-seq data from The Cancer Genome Atlas Pan-Cancer Initiative for primary and metastatic/recurrent lung adenocarcinoma, breast invasive carcinoma, and pancreatic adenocarcinoma tumors, as well as normal tissues. All primary tumor tissues exhibited significantly higher expression of *CEACAM5* compared to normal tissues. Because the TCGA data are derived from tissue RNA-seq while the protein data generated for this study are derived from plasma, they are not directly comparable. However, the TCGA expression results raise the possibility that elevated CEACAM5 in plasma may precede tumor metastasis.

The purpose of this analysis was to characterize protein outliers and dynamic protein changes in individuals prior to their diagnosis with cancer. This was made possible through deep phenotyping and biobanking in a “real-world” program of seemingly healthy individuals. This design results in a limited number of cases for any specific disease, but it does generate a large amount of longitudinal data for each individual *preceding* disease diagnosis. Although we observed elevated markers of metastasis years before diagnosis in some cases, no doubt our results would be strengthened by the addition of sample time points even *earlier* in the progression of our metastatic cancer cases. However, a much larger ‘healthy’ population tracked over a longer period of time would be needed to obtain these data. Tracking a high-risk population (e.g. individuals with known genetic or clinical risk factors) could be another effective strategy. Such deeply-phenotyped longitudinal datasets are beginning to emerge, and will progressively accumulate data for a growing number of participants who receive a cancer diagnosis.

Observing multiple proteins in a single individual that exhibit persistent outlier status or multiple proteins exhibiting rapid change over a short period of time suggests an underlying biological cause, rather than a technical explanation. Once we have established that a set of N-of-1 observations is likely biological in nature, the next task is to prospectively discern their significance in a larger study where we can estimate sensitivity and specificity in regards to disease prevalence in an appropriately chosen cohort matching the potential use case. As our understanding of human genetics and biological networks increases, combined with improved ability to measure thousands of phenotypes at regular intervals, our ability to prospectively infer disease transitions will certainly improve.

## Methods

### Study population

De-identified data from individuals in a commercially-available lifestyle intervention program (Arivale Inc., Seattle, WA) were collected from 2015 to 2018. The Arivale program involved health coaching on exercise, nutrition, stress management and other wellness goals. Individuals identified and voluntarily joined the program through commercial advertisement and/or verbal communication. Individuals who joined were required to be over the age of 18 and not pregnant, with no additional screening of participants. Demographic and proteomic data from a total of 79 individuals were included in the current study (Table 1, Supplemental Table S1). Out of the 79, ten individuals were designated as ‘cases’, who reported a cancer diagnosis following at least three visits in which biobanked plasma samples were collected. The additional 69 controls were chosen to match the 10 cancer cases using the following criteria: (1) At least 3 consecutive blood draws collected no more than 240 days apart; (2) Similar sex, age, and race distribution to cases; (3) No major disease transitions reported during the time period in which the measurements were collected.

All research was conducted in accordance to regulations and guidelines for observational research in human subjects. The research was performed entirely using de-identified and aggregated data of individuals who had signed a research authorization allowing the use of their anonymized data in research. The study was reviewed and approved by the Western International Review Board (Study Number 1178906). Western International Review Board found that this study met the requirements for a waiver of consent under 45 CFR 46.116(d).

### Sample collection

For each individual, blood collections were performed up to 3 times over a 12-month period at LabCorp facilities. Participants were asked to avoid alcohol, vigorous exercise, aspartame or monosodium glutamate 24 h prior to blood draw, and also to begin fasting 12 h in advance. Whole blood samples were collected in a 6.0 mL Royal Blue Top EDTA tube and centrifuged for at least 10 min. The plasma layer was transferred to a 2 mL polypropylene screw-capped transfer tube and frozen at – 80 °C. Frozen plasma samples were shipped to Brooks Life Sciences (Indianapolis, IN), and shipped in batch to the vendor.

### Proteomics

Plasma concentrations of proteins were measured using 13 ProSeek protein biomarker panels (Olink Biosciences, Uppsala, Sweden) at Olink facilities in Watertown, MA (Supplementary Table S2). The ProSeek method is based on the highly sensitive and specific proximity extension assay^7^, which involves the binding of distinct polyclonal oligonucleotide-labelled antibodies to the target protein followed by quantification with real-time quantitative polymerase chain reaction (rt-PCR).

### Median absolute deviation

To identify outliers in our proteomics data, we used median absolute deviation (MAD). This approach is more robust to the influence of outliers than the standard deviation, because it relies on the median of the data rather than the mean. MAD is the median of the absolute deviations from the data’s median value:

$$MAD = median\left( {\left| {X_{i} - \tilde{X}} \right|} \right)$$

where $\tilde{X} = median\left( X \right)$. We chose lower and upper cutoffs for outliers to be the value of the 1st and 99th percentile of MAD across all measured proteins, respectively. The value of the lower cutoff was − 3.755, and the value of the upper cutoff was 4.431. MAD values above 4.431 or below − 3.755 were considered to be outliers for this study. Proteins for which three longitudinal values were outliers within the same individual were defined as persistent outliers for that individual (Supplementary Table S3). We also created a ‘delta cutoff’, selected as the 99th percentile of $\left| {{\Delta }MAD} \right|$ between any two adjacent protein observations in the dataset. The value of the delta cutoff was 5.224. $\left| {{\Delta }MAD} \right|$ values greater than 5.224 between any two adjacent protein observations were considered to be ‘spikes’ in that protein.

### Resampling

We used resampling to estimate the significance of the CEACAM5 configuration of outliers (outliers in 7 observations split across 3 unique individuals). We resampled the data by randomly selecting 3 individuals without replacement from our population of 79 individuals, then randomly selecting 3 observations without replacement from each of those individuals (some individuals have more than 3 observations). We counted the number of outliers for each protein across these 9 observations. This process was repeated 5000 times to establish an empirical distribution of outliers for triplets of individuals. 54 out of 5,965,000 individual triplet-protein observations yielded 7 or more outliers, yielding an empirical p-value = 9.1e−6). See Supplementary Fig. S1. We used this empirical approach due to the limited amount of data that was available in which to observe longitudinal protein outliers. Future studies with much larger N may be able to establish a distribution of each configuration of outliers directly from the observed data.

### Statistical analysis

MAD and resampling analyses were performed in Python 3.6.6 using custom code. Figures were created using Seaborn 0.9.0. TCGA expression differences were modeled using Ordinary Least Square (OLS) linear regression models (Python statsmodels package), assessing the relationship between the batch normalized transformed expression of *CEACAM5* and the sample type (normal tissue, primary tumor), adjusting for age and sex.

### Validation analysis

RNA-seq data was extracted from The Cancer Genome Atlas Pan-Cancer Initiative on the Genomic Data Commons portal^11^. The data contained batch normalized and scaled transcript-aligned reads from RNASeqV2 mRNA data annotated with pertinent TCGA Barcodes, generated from the RNA-Seq by the Expectation Maximization (RSEM) package^22^. The data was extracted from the BigQuery table annotated ‘EBpp_AdjustPANCAN_IlluminaHiSeq_RNASeqV2_genExp_annot’.

Participants were subsequently filtered using the Pan-Cancer Atlas whitelist (table Auxiliary.Whitelist_ParticipantBarcodes), and the values for each gene were log transformed prior to analysis.

## Supplementary information

Supplementary file1

Supplementary file2
