# Supplementary material for: Untargeted longitudinal analysis of a wellness cohort identifies markers of metastatic cancer years prior to diagnosis
Source: Sci Rep. 2020 Oct 1;10:16275. doi: 10.1038/s41598-020-73451-z (PMC7529776; doi:10.1038/s41598-020-73451-z)
Supplement: Supplementary file 1 — Supplementary file1 [file 41598_2020_73451_MOESM1_ESM.pdf]

SUPPLEMENTARY INFORMATION: Untargeted longitudinal analysis of a wellness cohort identifies markers of metastatic cancer years prior to diagnosis

Andrew T Magis[1,*,†], *Noa Rappaport*[1], *Matthew P Conomos*[2], *Gilbert S Omenn*[1,3] *Jennifer C Lovejoy*[1], *Leroy Hood*[1,4], *Nathan D Price*[1,†]

[1]Institute for Systems Biology, 401 Terry Ave N, Seattle, WA 98109, USA
[2]Department of Biostatistics, University of Washington, Seattle, WA, USA
[3]Departments of Computational Medicine & Bioinformatics, Internal Medicine, Human Genetics, and School of Public Health, University of Michigan, Ann Arbor, MI, USA
[4]Providence St. Joseph Health, Seattle, Washington, USA
*Corresponding author
†Co-senior authors

SUPPLEMENTARY TABLES

Supplementary Table 1. Baseline characteristics of the cohort

**Characteristic**	**Total N=79**
Age, mean years (sd)	56.1 (11.7)
Males, no. (%)	41 (51.9)
Nonwhite, no. (%)	2 (2.5)
Number of blood draws per person, mean (range)	3.1 (3-6)
BMI, mean kg/m^2 (sd)	27.1 (5.8)
Current self-report of:	
High triglycerides, no. (%)	24 (30.4)
Osteoarthritis, no. (%)	13 (16.4)
Hypertension, no. (%)	10 (12.7)
Depression, no. (%)	8 (10.1)

Supplementary Table 2. Olink panels and number of proteins used for this study.

**Panel**	**# proteins**
Cardiometabolic	92
Cardiovascular 2	92
Cardiovascular 3	92
Cell Regulation	92
Development	92
Immune Response	92
Inflammation	92
Metabolism	92
Neuro-exploratory	92
Neurology	92
Oncology 2	92
Oncology 3	92
Organ Damage	92
**Total**	**1196**

Supplementary Table 3. All persistent outliers identified in this study across all samples and proteins. Provided in separate file.

SUPPLEMENTARY FIGURES

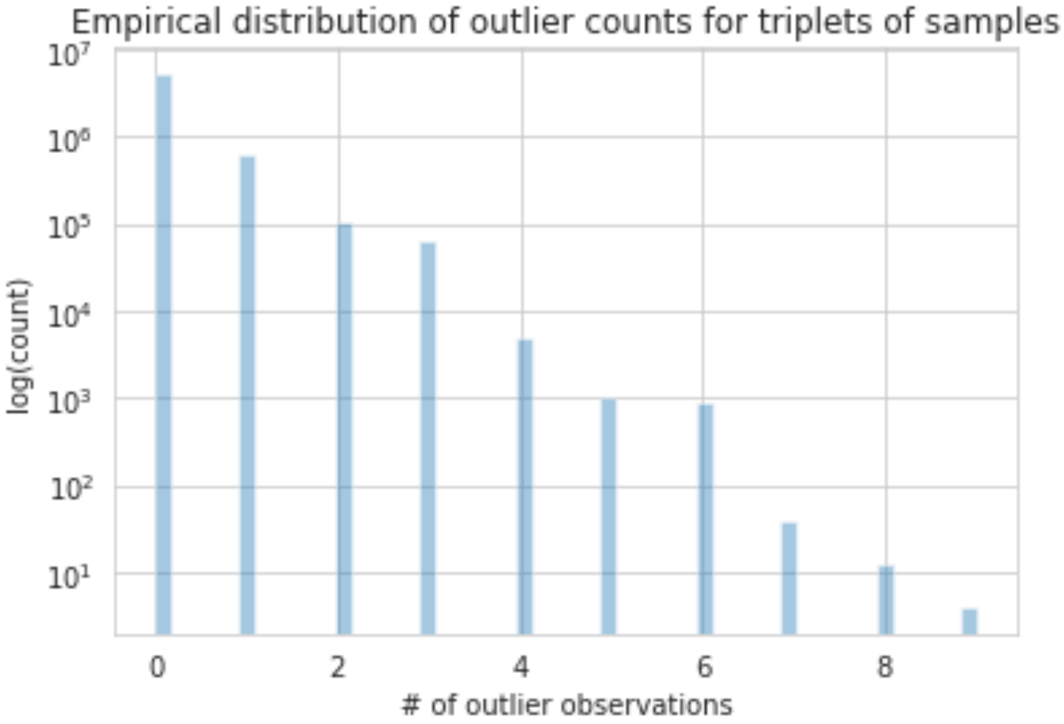

Supplementary Figure 1. Empirical distribution of outliers for resampled triplets of individuals with 3 samples each. The y-axis is on the log10 scale. The total number of observations of 7 outliers across 3 individuals was N=54, out of 5,965,000 combinations tested.

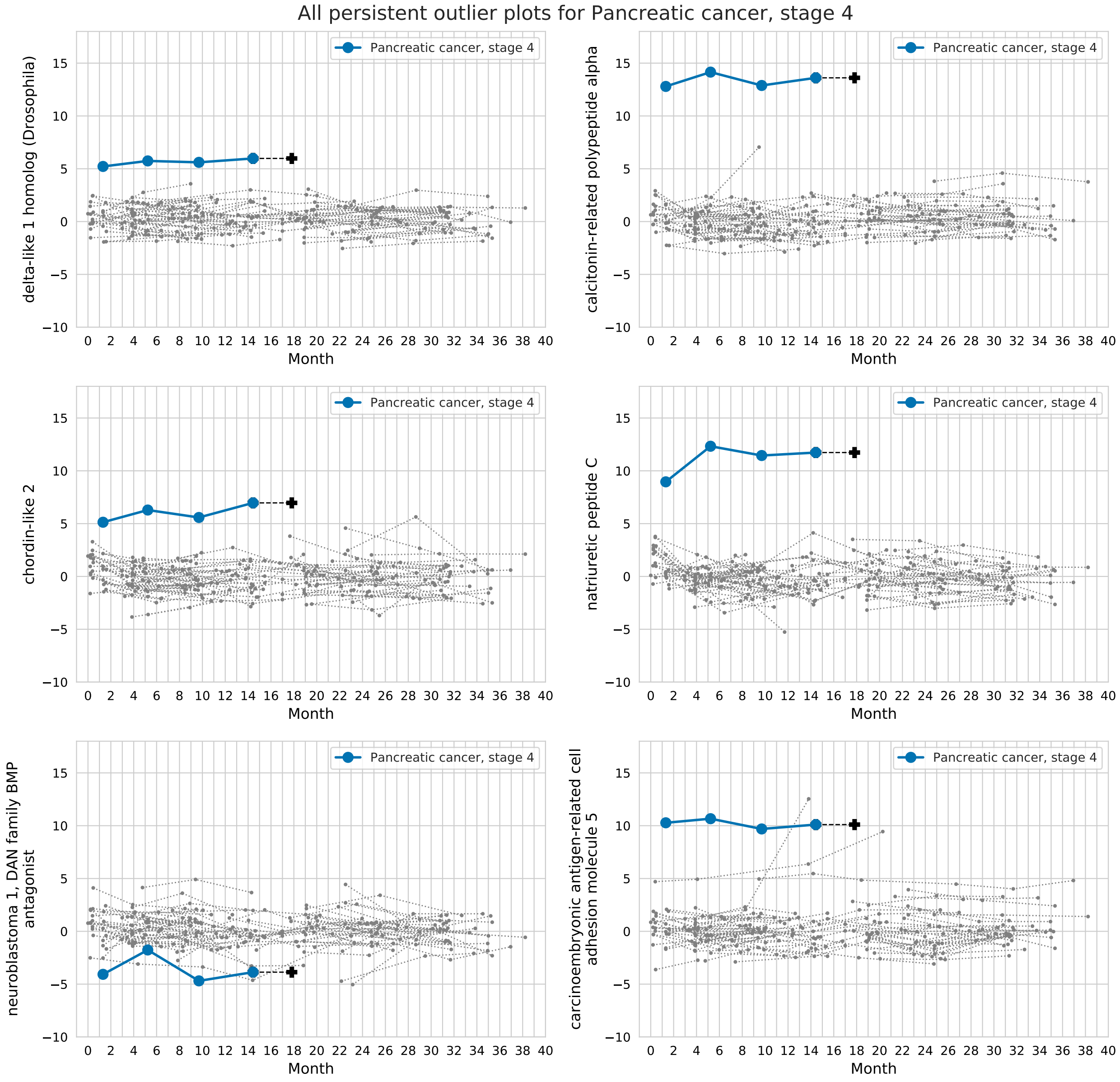

Supplementary Figure 2. All persistent outliers for Pancreatic cancer, stage 4

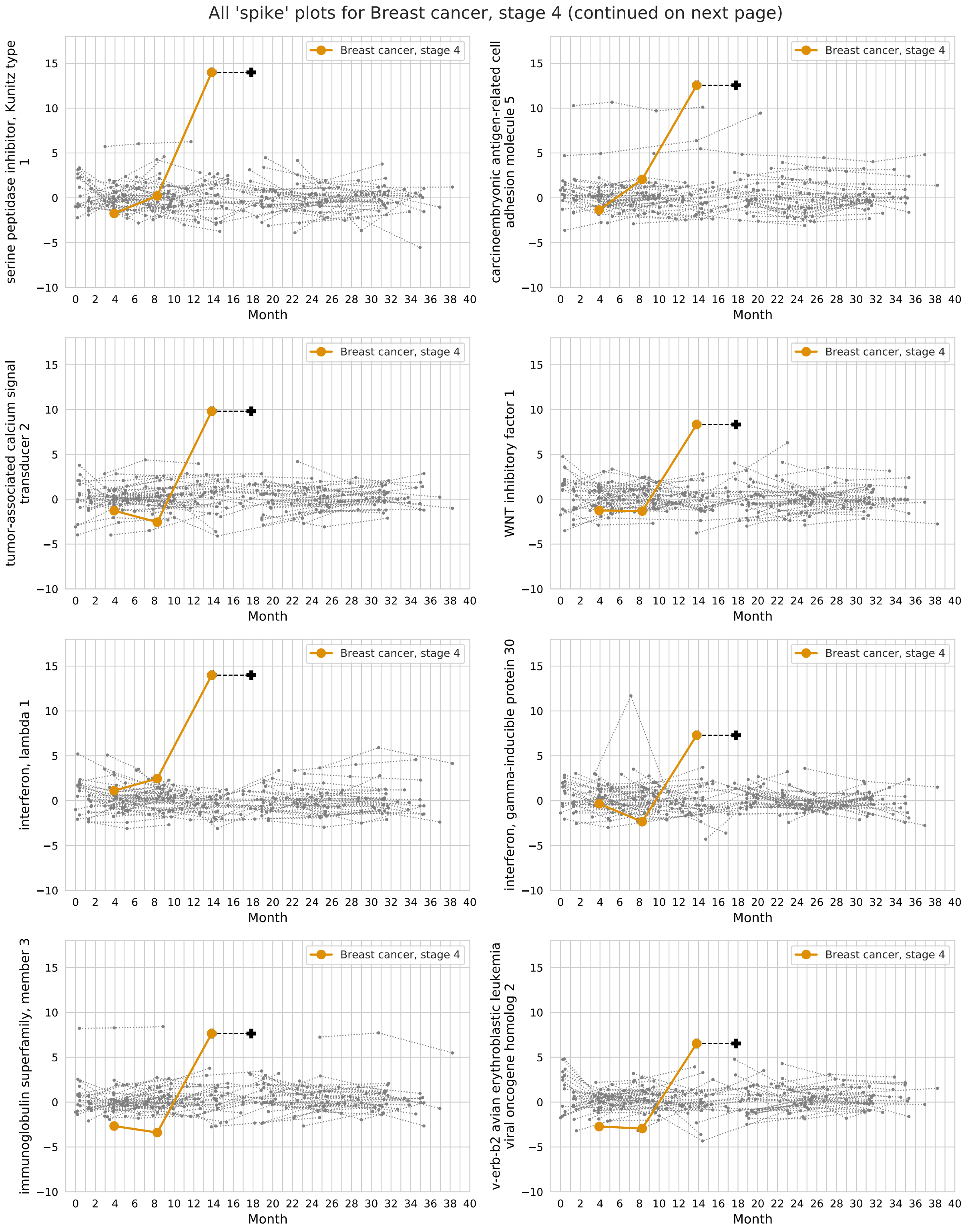

Supplementary Figure 3a. All 'spike' plots for Breast cancer, stage 4 (continued on next page). Values are sorted in descending order of $|\Delta MAD|$.

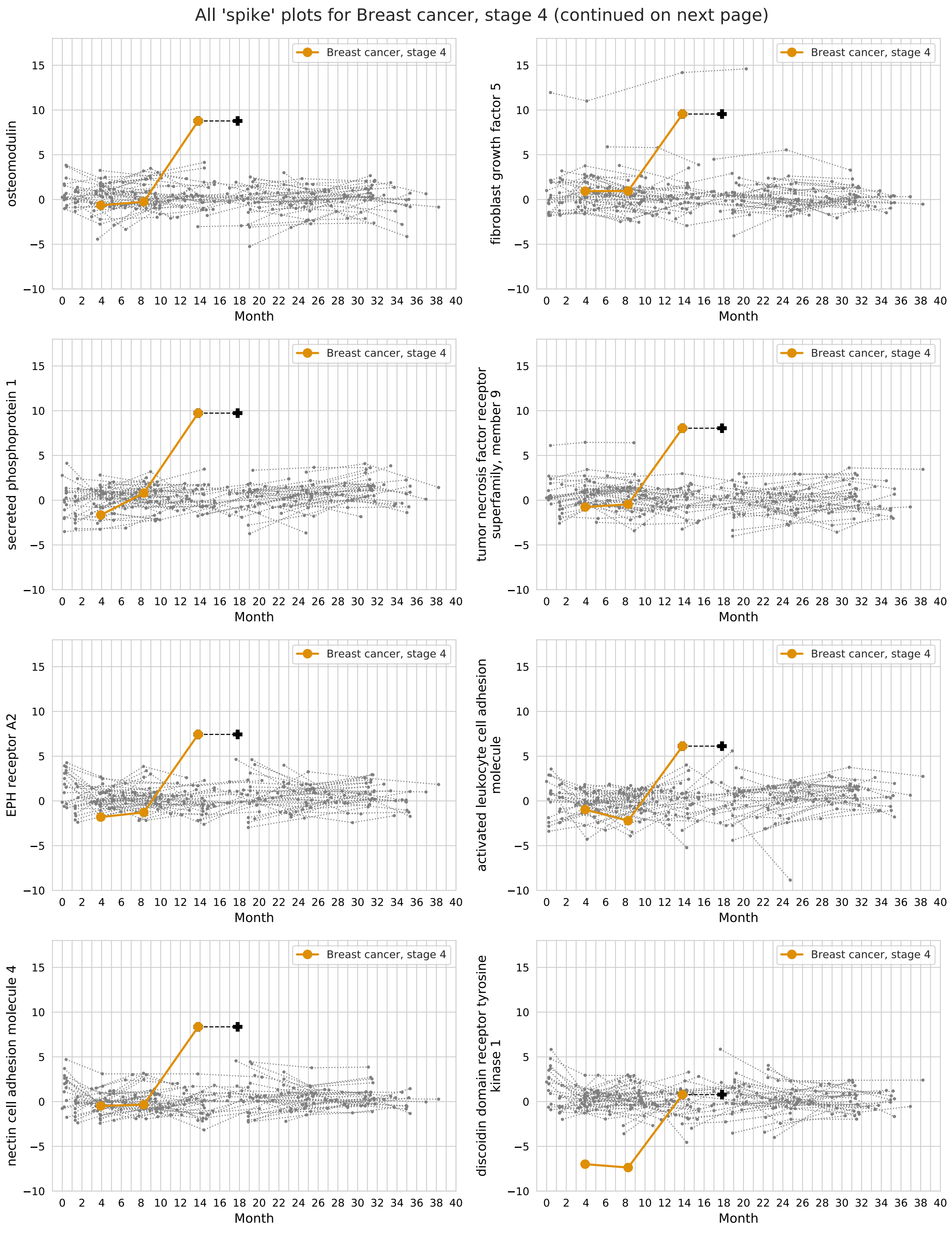

Supplementary Figure 3b. All ‘spike’ plots for Breast cancer, stage 4 (continued on next page). Values are sorted in descending order of $|\Delta MAD|$.

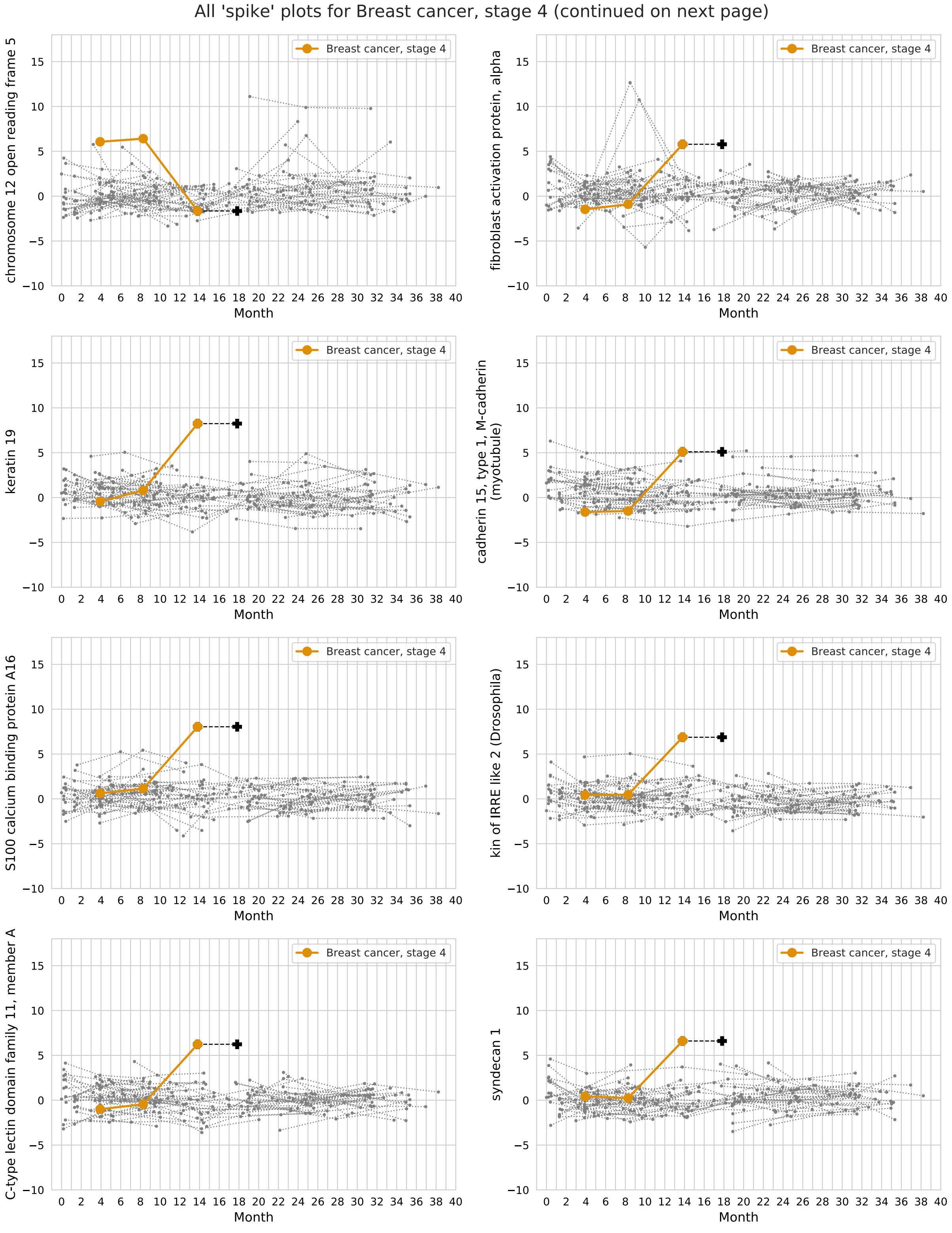

Supplementary Figure 3c. All 'spike' plots for Breast cancer, stage 4 (continued on next page). Values are sorted in descending order of $|\Delta MAD|$.

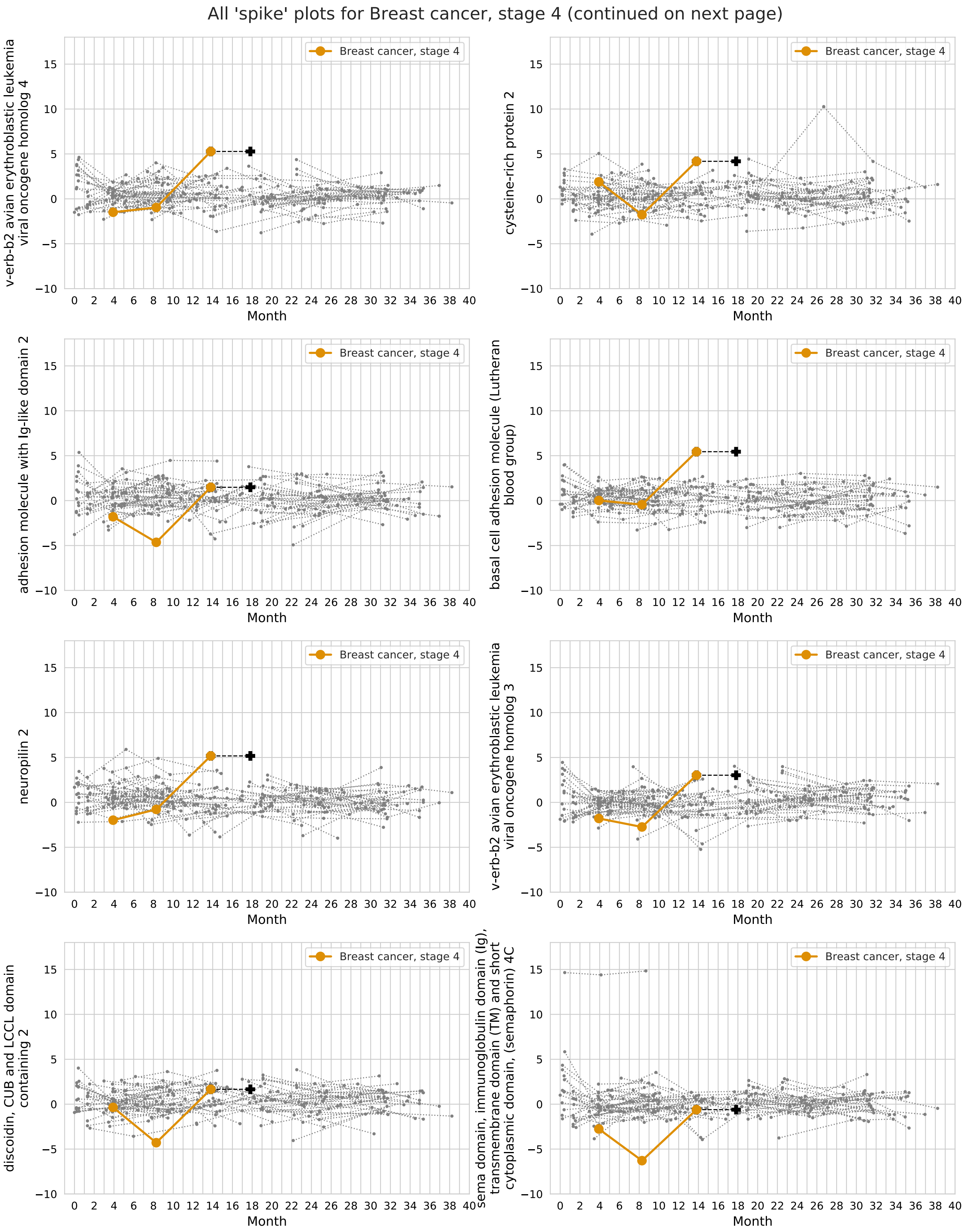

Supplementary Figure 3d. All 'spike' plots for Breast cancer, stage 4 (continued on next page). Values are sorted in descending order of $|\Delta MAD|$.

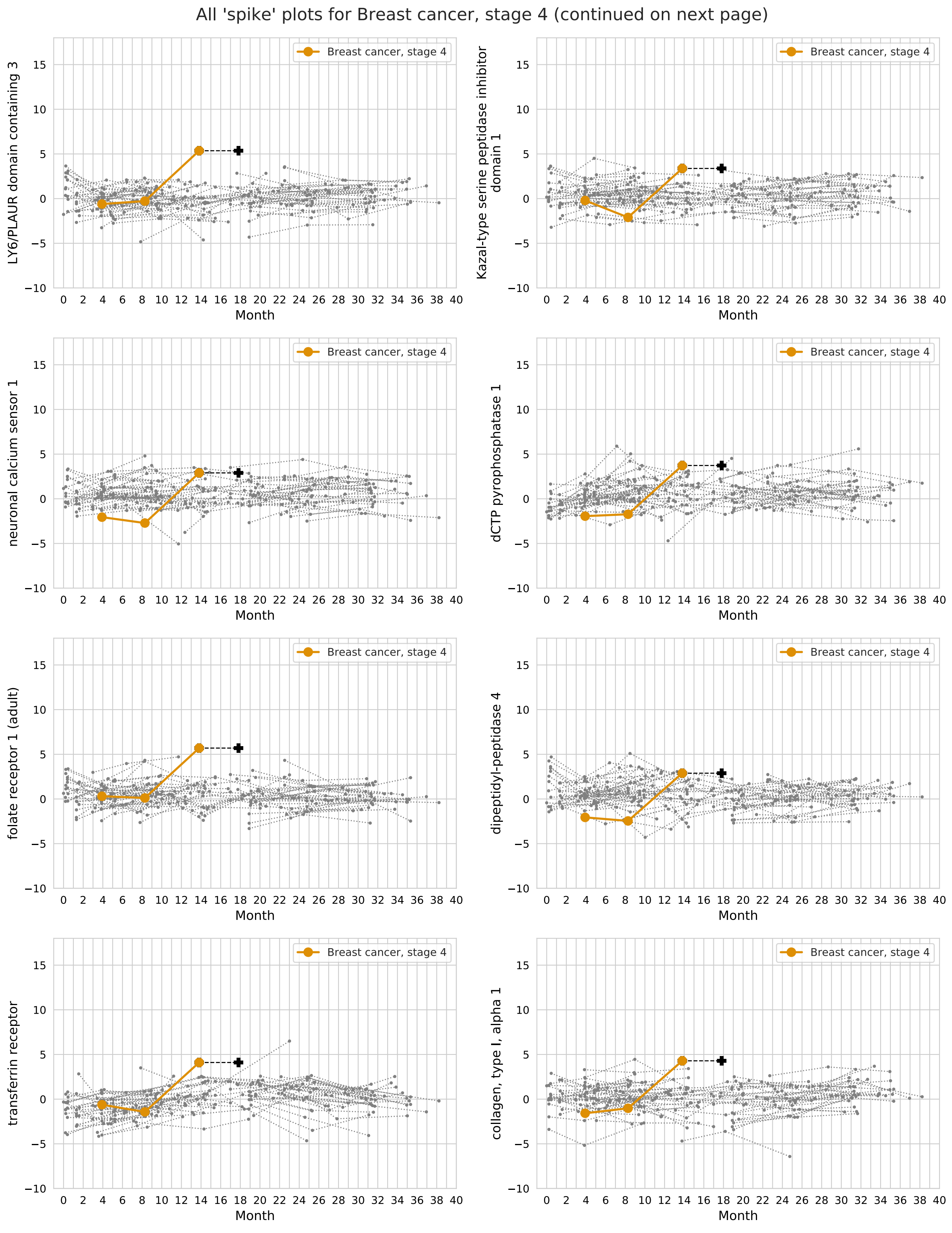

Supplementary Figure 3e. All 'spike' plots for Breast cancer, stage 4 (continued on next page). Values are sorted in descending order of $|\Delta MAD|$.

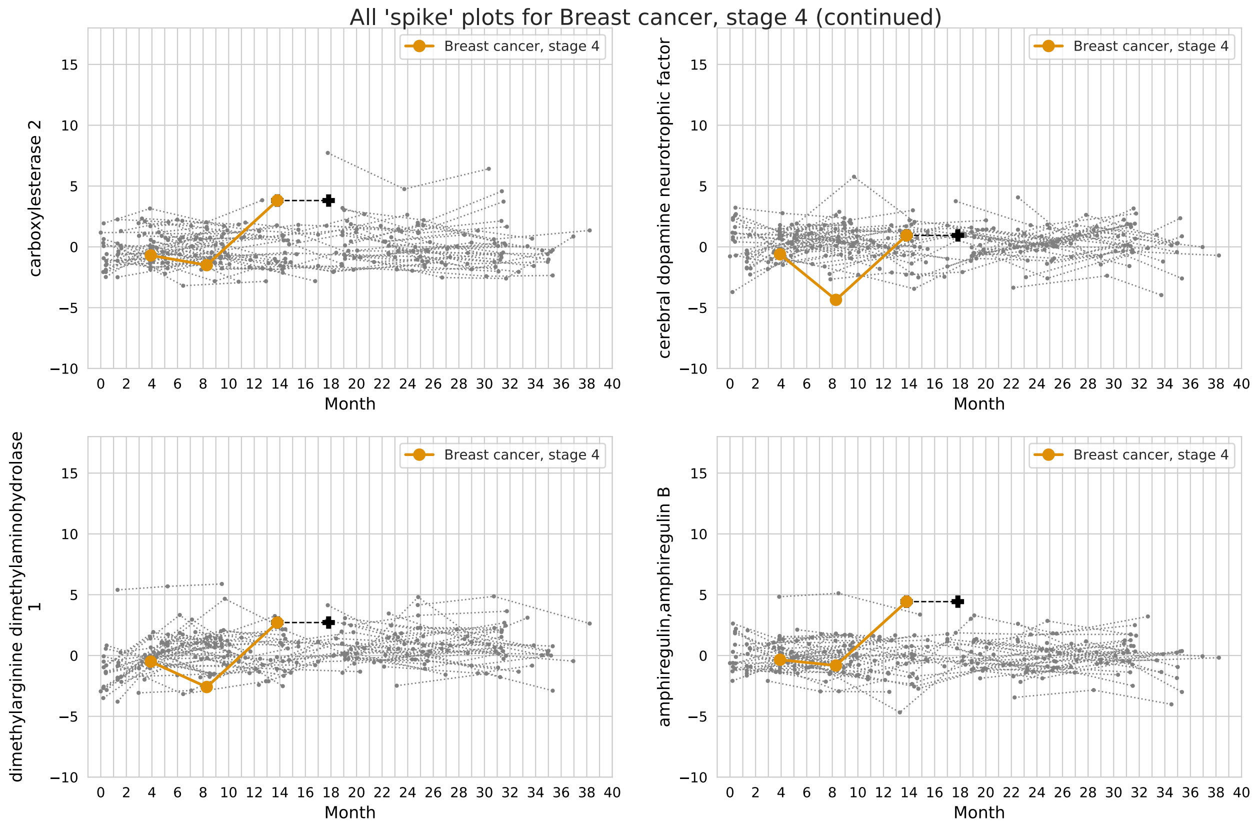

Supplementary Figure 3f. All 'spike' plots for Breast cancer, stage 4 (continued from previous page). Values are sorted in descending order of $|\Delta MAD|$.

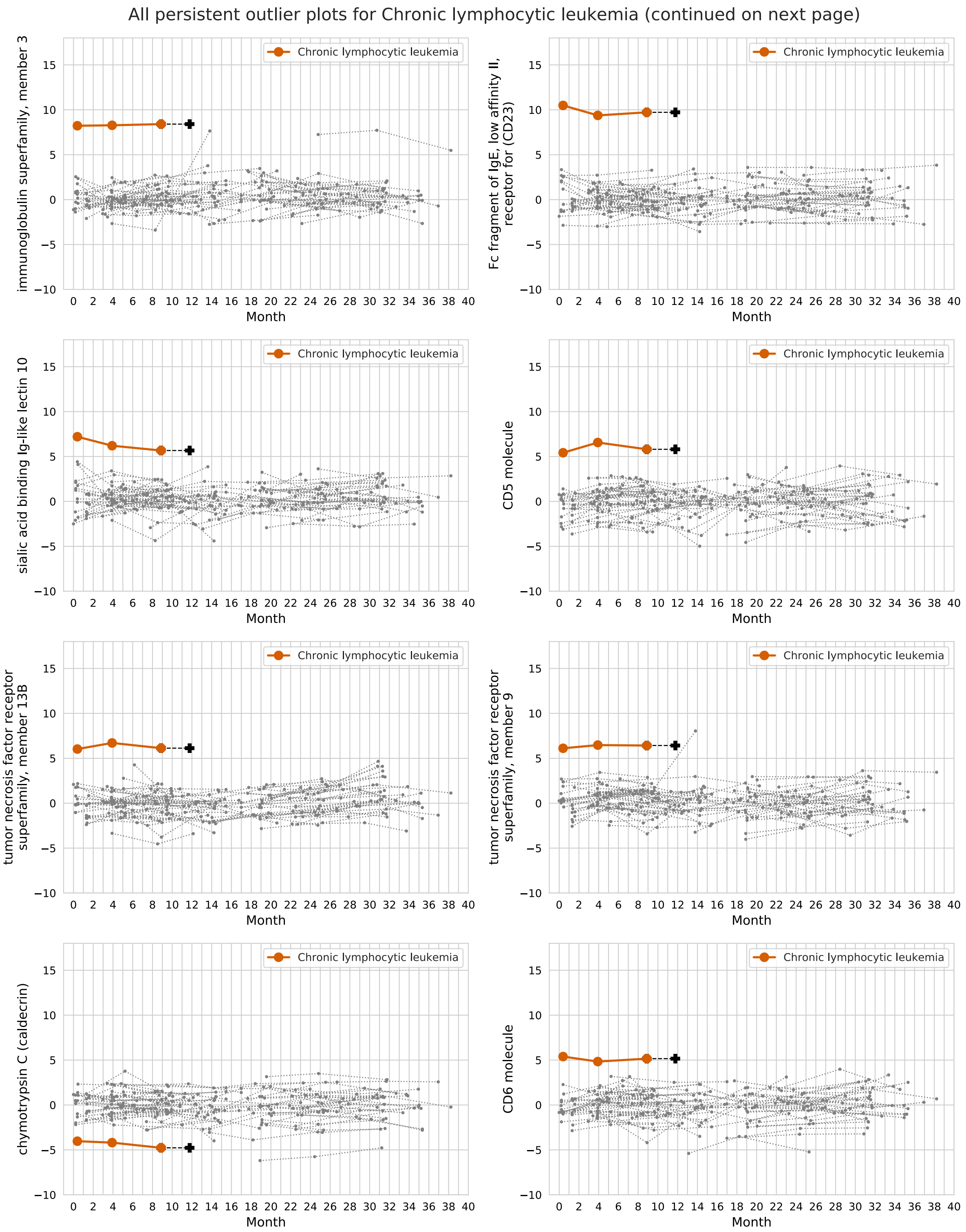

Supplementary Figure 4a. All persistent outliers for Chronic lymphocytic leukemia (continued on next page).

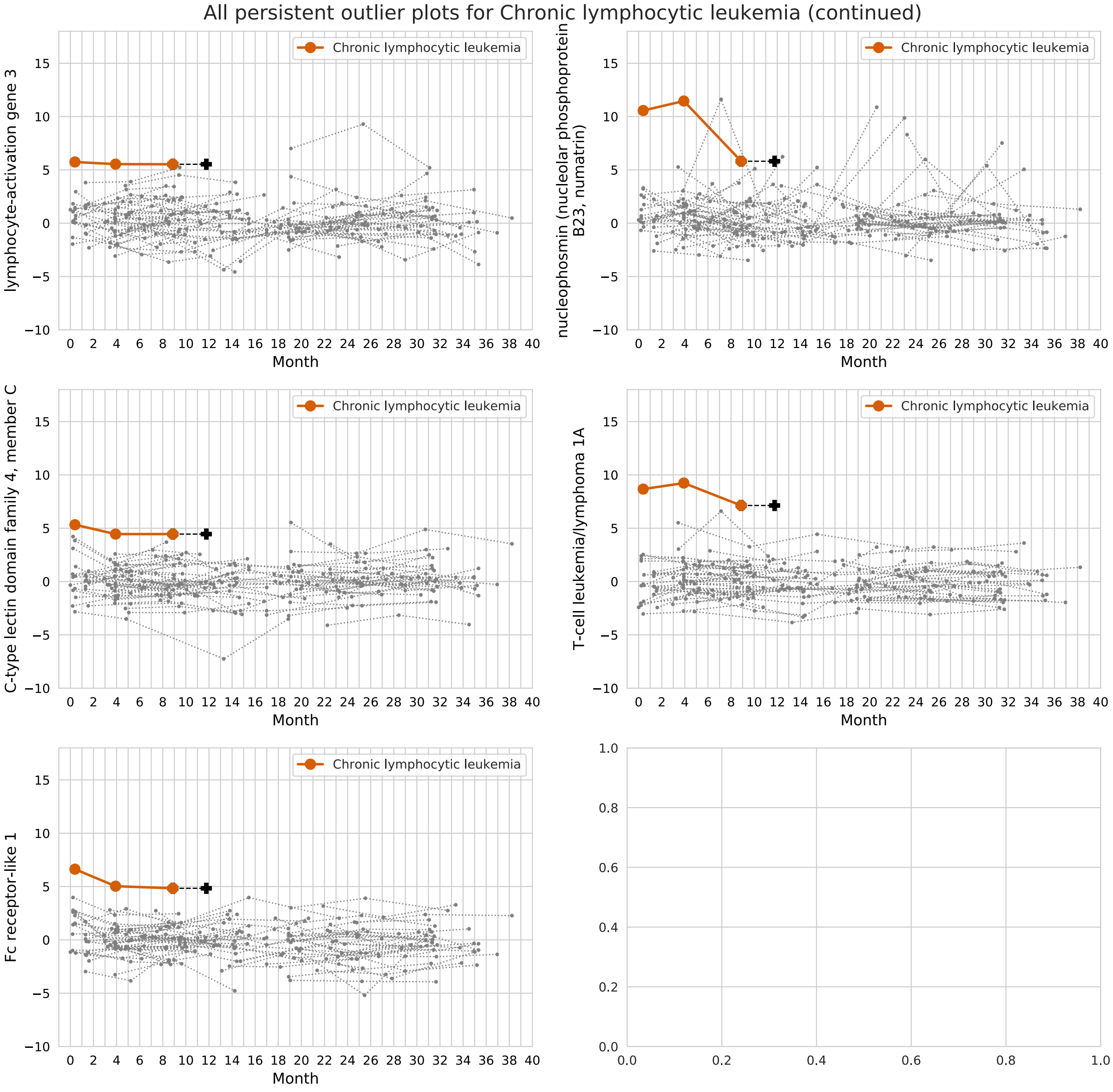

Supplementary Figure 4b. All persistent outliers for Chronic lymphocytic leukemia (continued from previous page).

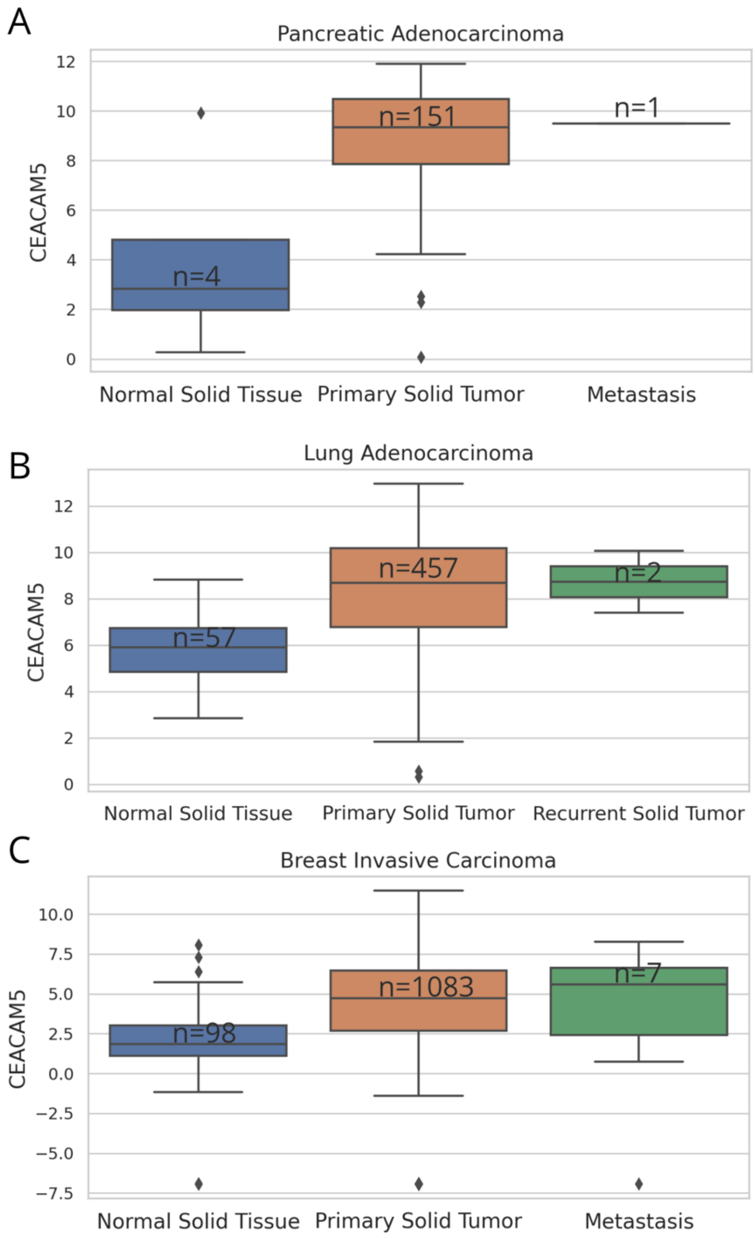

Supplementary Figure 5. RNA-seq expression levels of *CEACAM5* for normal, primary, and metastatic/recurrent pancreatic adenocarcinoma, lung adenocarcinoma, and breast invasive carcinoma, as well as normal tissues. The y-axis is batch-normalized and scaled transcript-aligned reads generated from RNA-Seq by the Expectation Maximization (RSEM) package. Data were obtained from The Cancer Genome Atlas Pan-Cancer Initiative. Box plots represent the interquartile range (25th to 75th percentile, IQR), with the middle line demarking the median; whiskers span 1.5 × IQR, points beyond this range are shown individually. Sample size for each group is noted above the median line.
